# Dual-task training is as effective as functional training on the functional fitness of older women: a randomized clinical trial

**DOI:** 10.1186/s12877-024-05204-w

**Published:** 2024-07-16

**Authors:** José C. Aragão-Santos, David G. Behm, Tatiana R. de Moura, Marzo Edir Da Silva-Grigoletto

**Affiliations:** 1 Department of Physical Education, Graduate Program in Health Sciences, Federal University of Sergipe, São Cristóvão, Brazil; 2 School of Human Kinetics and Recreation, Newfoundland and Labrador, Memorial University of Newfoundland, St. John’s, Canada; 3 Department of Morphology, Graduate Program in Health Sciences, Federal University of Sergipe, São Cristóvão, Brazil; 4 Department of Physical Education, Graduate Program in Physical Education, Federal University of Sergipe, São Cristóvão, Brazil

**Keywords:** Exercise, Multitasking behavior, Functional status, Physical fitness, Health, Aging

## Abstract

**Background:**

The interest in approaches that improve older individuals’ functional fitness and autonomy is increasing. However, the effects of dual-task training on older women’s functional fitness and the comparison with the functional training approach are unclear. Therefore, we compared dual-task and functional training on the functional fitness of older women and the effects of three months of detraining.

**Methods:**

Sixty-one women performed 16 weeks of dual-task training or functional training. The functional fitness was measured pre-, post-training, and post-detraining, based on the ability to put on and take off a t-shirt, evaluating the mobility of the upper limb, standing-up from the prone position measuring the global functionality, five times sit-to-stand test to assess the lower limbs muscle power, timed up and go to measure the dynamic balance and agility, gallon-jug shelf-transfer to evaluate the global functionality emphasizing the upper limbs and 10 m walk test to analyze the gait ability.

**Results:**

Dual-task training and functional training generally provided significant small to moderate magnitude performance increases in the put on and take off a t-shirt (dual-task training: d = 0.35 / functional training: d = 0.49), five times sit-to-stand test (dual-task training: d = 0.41 / functional training: d = 0.77), timed up and go (dual-task training: d = 0.34 / functional training: d = 0.78), and gallon-jug shelf-transfer (dual-task training: d = 0.76 / functional training: d = 0.82). Only the functional training improved the 10 m walk test (d = 0.32; *p* = 0.013), and both groups did not change the standing-up from the prone position performance. After the detraining period, both groups kept the adaptations for the gallon-jug shelf-transfer and five times sit-to-stand test. At the same time, only the dual-task training maintained the adaptations for the put on and take off a t-shirt and the functional training for the timed up and go.

**Conclusion:**

Sixteen weeks of dual-task and functional training are similarly effective in improving older women’s functional fitness, maintaining their benefits even after three months of detraining.

**Trial registration:**

RBR-10ny848z (https://ensaiosclinicos.gov.br/rg/RBR-10ny848z).

## Background

Declines in functional fitness and cognitive health accompany aging and are exacerbated by reduced physical activity [1, 2]. In addition, menopause-related alterations make women more susceptible to these harmful effects [3]. Among these declines, the reduction in gait speed [4], dynamic balance and agility, and muscle power [5] play an important role in personal autonomy reduction and increased injury incidence (e.g., increased falls and slips with decreased balance, agility, and power). Hence, the interest in treatments that attenuate these declines and concomitantly improve the functional fitness of the older population is increasing [6].

Several guidelines suggest that physical exercise improves older individuals’ general health [7, 8]. Generally, a common aspect in the literature is the relevance of strength training in preventing sarcopenia and age-related effects [8]. Recently, Izquierdo et al. [7] suggested applying multi-component training as a suitable approach for stimulating the different functional fitness aspects and attending to the necessities of older adults. In this sense, our research group has put effort into understanding functional training (FT) implications for older adults since it is a multi-component approach that emphasizes the activities of daily living and explores the specificity principle [9, 10].

FT uses everyday life patterns such as push, pull, carry-on, and squat, besides combining different movement patterns, acceleration, and deceleration, to explore complexity as a way of progression [11]. Despite the several FT benefits, some people cannot perform moderate to high-intensity exercises due to health problems or do not like weight lifting. Hence, adhering to training programs and getting health benefits is more challenging. Thus, another approach for older adults is using dual-task training (DT) to make the exercise more challenging without emphasizing the external overload principle [12].

The DT combines motor and cognitive stimuli sequentially or simultaneously [13]. The sequential approach dedicates one session part to the motor activities and another to the cognitive tasks [13], similar to the concurrent resistance and endurance training approach. In turn, the simultaneous approach combines both stimuli (motor + cognitive) in the same session [13], which is more similar to the FT protocols stimulating multiple physical capacities concomitantly. Also, simultaneous DT seems to provide more benefits for older people than sequential DT, besides solving the problem of the optimal interval between the motor and cognitive stimuli and the time consumption present in the sequential approach [14]. However, most studies explore the effects of this type of training on executive functions and cognitive variables [15] without providing an extensive DT protocol and investigating the effects on functional tasks similar to activities of daily living and the detraining effects.

Thus, we compared DT and FT and examined the effects of detraining on the functional fitness of older women. Due to the overload and specificity principles, we hypothesized that DT would promote functional fitness benefits, but the FT would provide superior effects. Additionally, the detraining results would show that FT is more effective in maintaining the adaptations due to the large magnitude of FT-derived effects due to the muscle power stimuli.

## Methods

### Study design

**Fig. 1:**
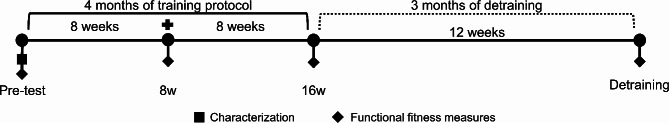
Research design. *Note.*w: week

We conducted a single-blinded randomized clinical trial with two arms between July 2022 and January 2023. Sixteen weeks were dedicated to applying the training protocols (DT vs. FT), and we conducted a follow-up period of three months without training sessions (Detraining). During the study period, four assessments were taken: the first assessment at the beginning (Pre-test), the second in the middle (8 weeks), the third at the end of the intervention period (16 weeks), and the last assessment after three months of detraining (Fig. 1). All interventions were conducted at the university’s sports facilities during the morning. The measurements and the data analysis were performed by researchers who were not aware of the protocol performed for each participant. This clinical trial was registered on the Brazilian Registry of Clinical Trials (RBR-10ny848z) and followed the Consort guidelines to report clinical trials [16].

### Participants

The participants were recruited through social media, advertisements at the religious and health centers in the community, and leaflets around the university. To participate in the study, the participants were required to be 60 years or older, able to walk independently, achieve at least 21 points on the Montreal Cognitive Assessment [17], and be free of cardiovascular or musculoskeletal diseases (not controlled or treated) that could prevent participation in physical exercise interventions.

The sample size was calculated using the General Linear Mixed Model Power and Sample Size (http://glimmpse.samplesizeshop.org/) [18] based on the gallon-jug shelf-transfer results found by Aragão-Santos et al. [9]. Specifically, we adopted a power of 90%, an alpha error of 0.05, the means, and the polled standard deviation to estimate the sample size for a generalized linear mixed model analysis. We estimated a need for 46 participants. After considering the possible sample losses during the intervention, we added 20% to the total, achieving a minimum of 56 participants.

After the recruitment, 149 older women demonstrated interest in participating in the study, and 61 met the eligibility criteria. The eligible participants were randomly allocated to the FT or DT group at a ratio of 1:1 based on the results of the gallon-jug shelf-transfer test. Specifically, the participants were sequentially numbered based on the test results, random numbers were attributed to each participant, and blocks of two participants were created. Afterward, the participants with the smaller random number in each block were allocated to the FT, and the other participants were assigned to the DT. Since the participants were aware of the training protocols, they were allowed to change according to the participants’ preferences after the randomization to maximize adherence to the training program.

### Collection processes

#### General information

At first, the participants were evaluated by an interview for the eligibility evaluation, and general information was recorded, such as age, body mass, and height. For the performance tests, all the measures were performed by professionals experienced with the collection procedures and always during the morning. The evaluators were blinded to the protocols performed for each participant. Each test had a familiarization attempt to guarantee the participants’ comprehension and two more attempts to record the results with a 2-minute rest interval between attempts. The professionals provided verbal encouragement during the test execution and, if necessary, additional explanations. Every test was time-based (i.e., performed as rapidly as possible) and recorded using a stopwatch (Google LLC), so the participants were asked to perform the tests as fast as possible, and the shorter performance time was used for the subsequent analysis. Each test is described below.

#### Put on and take off a t-shirt (PTS)

This test evaluated mobility, coordination, and agility, focusing on the upper limbs used to put on and take off a t-shirt as quickly as possible. The participants used a t-shirt according to their size, choosing between small, medium, large, or extra-large, and the size chosen was used in both moments of evaluation. The participant started the test holding the t-shirt in her dominant hand. After the evaluator’s command (“Go!”), the participants put on the t-shirt completely, removed it, unraveled it, and held it in its initial position. The trial was invalidated when the participant did not wear the t-shirt completely, and another trial was performed [19].

#### Standing-up from the prone position (SPP)

The participant used her global strength and core stabilization muscle in this test. The participant initiated the test in a ventral decubitus position. Next, after the evaluator’s command (“Go!”), she would get up and keep the standing position. Additionally, an assistant stayed close to the participant in case of imbalance and fall risk [20].

#### Five times sit-to-stand test (FTSST)

This test evaluated the muscle power of the lower limbs indirectly. The participant began the test seated with her arms crossed over her chest. After the evaluator’s command (“Go!”), she would stand up five times consecutively with her arms crossed over her chest. If the participant did not touch the seat, the trial was invalidated, and another trial was performed [21].

#### Timed up and go (TUG)

The participant was required to use her agility and dynamic balance in this test. So, the participant was asked to initiate the test seated on a chair with her arms relaxed by the side. After the evaluator’s command (“Go!”), she would get up and walk by three meters, go around a cone, return to the seat, and sit again. If the participant kicked or bumped into the cone, the test was invalidated, and another trial was performed [22].

#### Gallon-jug shelf-transfer (GJST)

This test required the participant’s upper limb strength, agility, and coordination. After the evaluator’s command (“Go!”), the participant should move sequentially a gallon jug (3.9 kg) each time from a lower shelf at the patella height to an upper shelf at shoulder height. The participant should hold the gallon jug using her dominant hand and use another hand only to balance it. Also, the participant was required to keep her position lateral to the shelf. If the participant used two hands to hold the gallon jug or changed her position to the shelf (i.e., directly facing the shelf), the trial was invalidated, and another was performed [23].

#### 10 m walk test (W10m)

The participant was required to use her walking ability and speed for this test. After the evaluator’s command (“Go!”), the participant was asked to walk along a course of 14 meters. Specifically, two meters were used for acceleration, and two more were used for deceleration. Thus, the intermediate 10 m were used to register the time to perform the test and estimate the walking speed. The attempt was not considered if the participant ran during the test and another trial was conducted [24].

### Training protocols

For both groups, forty-eight sessions were performed (thrice weekly for 16 weeks). Each session lasted approximately 50 min and was set up in five parts that differed between groups. The training progression occurred every four weeks to promote variability in the stimulus and keep the training challenging. Also, during the detraining period, the participants were advised to keep their usual daily activities.

#### Dual-task training

The first part focused on joint mobility and stability combined with working memory and cognitive flexibility stimulus, lasting 10 min. Specifically, it was required that the participants tell fruit names, and the next participant should say a different fruit until all the participants said one fruit name. The second part stimulated static and dynamic balance combined with object manipulation, such as balls and sticks, for 15 min. Additionally, the participants were asked to keep the position (i.e., bipedal or unipedal) or walk alternating in tandem stance while counting up threes (i.e., 3, 6, 9, 12…), stimulating inhibitory control and working memory. The third part consisted of coordinative patterns or lateral displacements associated with a visual or sound command to change the pattern, stimulating the working memory for 15 min. The fourth part focused on reaction time to pass a ball and was associated with cognitive flexibility, lasting six minutes. Namely, the participants should say a color to pass the ball, and accordingly, with the command, the ball should be passed in different ways (i.e., above the head, under the legs, or by the side). The last part was used to warm down with breathing exercises for four minutes.

The intensity was monitored using the perceived effort rate due to the training protocol’s characteristics. The exercises progressed based on the variation between bipedal and unipedal positions or the space available for the displacements [25]. Also, the cognitive stimulus varied based on the type of word that should be said by the participant (i.e., fruit, class, city, country), the interval in counting activities, backward counting, or increasing the number of commands available in the tasks. Four professionals monitored the participants and guaranteed that the training protocol was applied correctly. Detailed information about the exercises and variations used is shown in Table 1.

**Table 1:** Dual-task training protocol

Parts	Activity	Weeks 1–4	Weeks 5–8	Weeks 9–12	Weeks 13–16
**Mobility/** **stability/** **evocation** **Total time = 10 min**	N/A	Two movement patterns for the main joints (glenohumeral, hip, and ankle) + fruit names evocation	Three movement patterns for the main joints + city names evocation	Three movement patterns for the main joints + isometric squat + food names evocation	Four movement patterns for the main joints + isometric squat + country names evocation
**Balance/** **handling** **Total time = 15 min**	Bipedal	Feet together + ball transfer from one hand to the other	Semi-tandem + single-handed ball movement from top to bottom and bottom to top	Tandem + ball transfer from one hand to the other	Tandem + balance the ball on the palm of the hand, moving it from left to right
	Unipedal	Using a balance aid stick + facing a partner + naming a fruit and the partner responding with the name of another fruit without repetition	Without the aid stick + facing a partner + naming a city and the partner responding with the name of another city without repetition	Holding a ball with one hand + facing a partner + naming a city, and transferring a ball from one hand to the other + the partner that should respond with the name of another city in the same way	Holding a ball with one hand + facing a partner + naming a city, and passing the ball to the partner that should respond with the name of another city and pass the ball back
	Walking	Straight line walking over a 20 centimeters line + balancing a horizontal stick	Straight line walking over a 10 centimeters line + balancing a horizontal stick	Straight line walking over a 10 centimeters line + transferring a ball from one hand to the other	Straight line walking over a 10 centimeters line + balance the ball on the palm of the hand
**Coordination/** **reaction time** **Total time = 15 min**	Without displacement	Stationary march + 1, 2, stop + 1, 2, 3, stop	Stationary march + 1, 2, stop + 1, 2, 3, stop + visual command to alternate between 1,2, stop and 1, 2, 3, stop	Stationary march + verbal command to stop	Stationary march + visual command to stop
	With displacement	Laterally	Laterally + arms opening and closing horizontally	Laterally + arms opening and closing horizontally and vertically	Laterally + arms opening and closing horizontally, vertically, and diagonally
	Handling	In-line formation + passing the ball laterally + naming fruits	In-line formation + passing the ball over the head and the next person in line passing the ball between the legs + naming countries	In-line formation + passing the ball over the head or between the legs accordingly to verbal command + counting in increments of 3	In-line formation + passing the ball over the head or between the legs accordingly to visual command + counting in increments of 7
**Flexibility** **Total time = 6 min**	N/A	2 sets of 10 s for the main muscles	3 sets of 10 s for the main muscles	2 sets of 20 s for the main muscles	3 sets of 20 s for the main muscles

*Note* N/A = not applicable

#### Functional training

The first part focused on joint mobility and stability, coordination, and pre-activation for six minutes. The second was dedicated to stimulating agility and muscle power based on coordinative activities for 15 min. The third explored strength exercises focusing on basic patterns such as pull, push, transportation, and squat, trying to emulate everyday life patterns lasting 20 min. The fourth used interval running to stimulate cardiorespiratory adaptations for five minutes. Finally, the fifth part was used to warm down with breathing exercises for four minutes.

The training intensity was based on the work and rest ratio or density. Namely, we started the intervention by applying a proportion of 1 to 2 (e.g., for 30 s of work and 60 s of rest). Based on the velocity specificity training to promote power adaptations [26], the participants were asked to perform the actions as fast as possible during the concentric phase of the movements. The load was adjusted based on the projected number of repetitions range, between 8 and 12 repetitions. Thus, if the participant performed more than 12 repetitions, the load was increased by 5%, and the external load was reduced in the same proportion if less than eight repetitions were performed. The bodyweight exercises were adjusted based on biomechanical changes [25]. Six professionals monitored the participants and ensured the proper protocol application. Detailed information about the performed activities and training load applied are shown in Table 2.

**Table 2:** Functional training protocol

Parts	Activity	Weeks 1–4 / D = 40s:40s	Weeks 5–8 / D = 40s:30s	Weeks 9–12 / D = 40s:20s	Weeks 13–16 / D = 40s:15s
**Mobility/** **stabilization/** **reaction time** **Total time = 6 min**	N/A	Two movement patterns for the main joints (glenohumeral, hip, and ankle) + frontal displacement + high knee skipping	Three movement patterns for the main joints + frontal displacement + high knee skipping + change of direction for skipping	Three movement patterns for the main joints + lateral displacement + air jump rope + reaction time for changing direction	Three movement patterns for the main joints + lateral displacement + air jump rope + change of direction + reaction time for squat or step
**Power/** **agility/** **coordination** **Total time = 15 min**	Ladder agility patterns	“Two-foot run”, “One-sided icky shuffle”, and “In, in, out, out”	“Ladder taps”, “Two-sided icky shuffle”, and “Two-sided icky shuffle touching a cone”	“Two-foot lateral run”, “One-footed lateral in, in, out, out”, “Two-footed lateral in, in, out, out”	“Two forward, one back”, “Straddle squat hops”, “Straddle squat hops laterally”
	Battle rope	Bilateral up and down waves	Unilateral up and down waves	Bilateral side-to-side waves	Inside and outside circles
	Medicine ball patterns	Slams	Hip drive and bilateral press	Hip drive and unilateral press	Same-side rotational throw
	Step patterns	Frontal step up and down	Frontal jump onto the step up and step down	Lateral step up and down	Lateral jump onto the step up and step down
	Displacements using cones	Forward shuffle between cones	Forward weave-around cones	Lateral shuffle between cones	Lateral weave-around cones
**Strength** **Total time = 20 min**	Kettlebell deadlift	Conventional	Side lunge unilateral	Suitcase unilateral	Suitcase alternating arms
	Goblet squat	Conventional	Kettlebell pick-up and set-down	Kettlebell pick-up and set-down, finishing on toes	Unilateral shoulder-loaded
	Farmer’s walk	Bilateral	Unilateral	Unilateral alternating arms	Bilateral with asymmetric weights
	Rowing	Neutral grip	Neutral grip with more inclination	Supinated grip	Pronated grip
	Chest press	Bilateral standing with elastic band	Bilateral standing with elastic band and knee raise	Unilateral standing with elastic band	Unilateral standing with elastic band and knee raise
**Cardiorespiratory fitness** **Total time = 5 min**	Relay race	Conventional	Zigzag pattern between cones	Zigzag pattern laterally between cones	Zigzag pattern laterally around cones

*Note* D = density; N/A = not applicable

### Statistical analysis

Collected data were analyzed using the statistical software Jamovi (The Jamovi Project, Jamovi, Version 2.3.18.0 [27], retrieved from https://www.jamovi.org). We present all continuous descriptive data as estimated marginal means and the 95% confidence interval and categorical data as absolute and relative frequency. We compared the groups at the beginning of the study for the characterization data with an independent t-test (continuous variables) and a chi-square test (categorical variables). Due to the repeated measures design, we used a generalized linear mixed model for the inferential analysis.

Based on the data distribution, QQ-plot graphs, and Akaike information criterion, we analyzed the data using a Gamma distribution model that better fits asymmetric data. We defined the groups as the inter-subject effect (FT and DT), the time as the intra-subject effect (i.e., Pre, 8-weeks, 16-weeks, and Detraining), and the interaction effect (group × time) as fixed effects. Besides, we set up the participants’ intercepts as a random effect to address individual variations in the repeated measures model.

If one or more fixed effects were statistically significant, we performed the post hoc pairwise comparisons (Bonferroni adjustment) to identify the differences between pairs. We set up the significance at *p* < 0.05 for all analyses. Additionally, we calculated the Cohen’s d effect size as a difference in the mean values between pre- and post-training divided by the pooled SD. ES of 0.00–0.19 was considered trivial, 0.20–0.49 was small, 0.50–0.79 was moderate, and ≥ 0.80 was large [28].

## Results

**Fig. 2:**
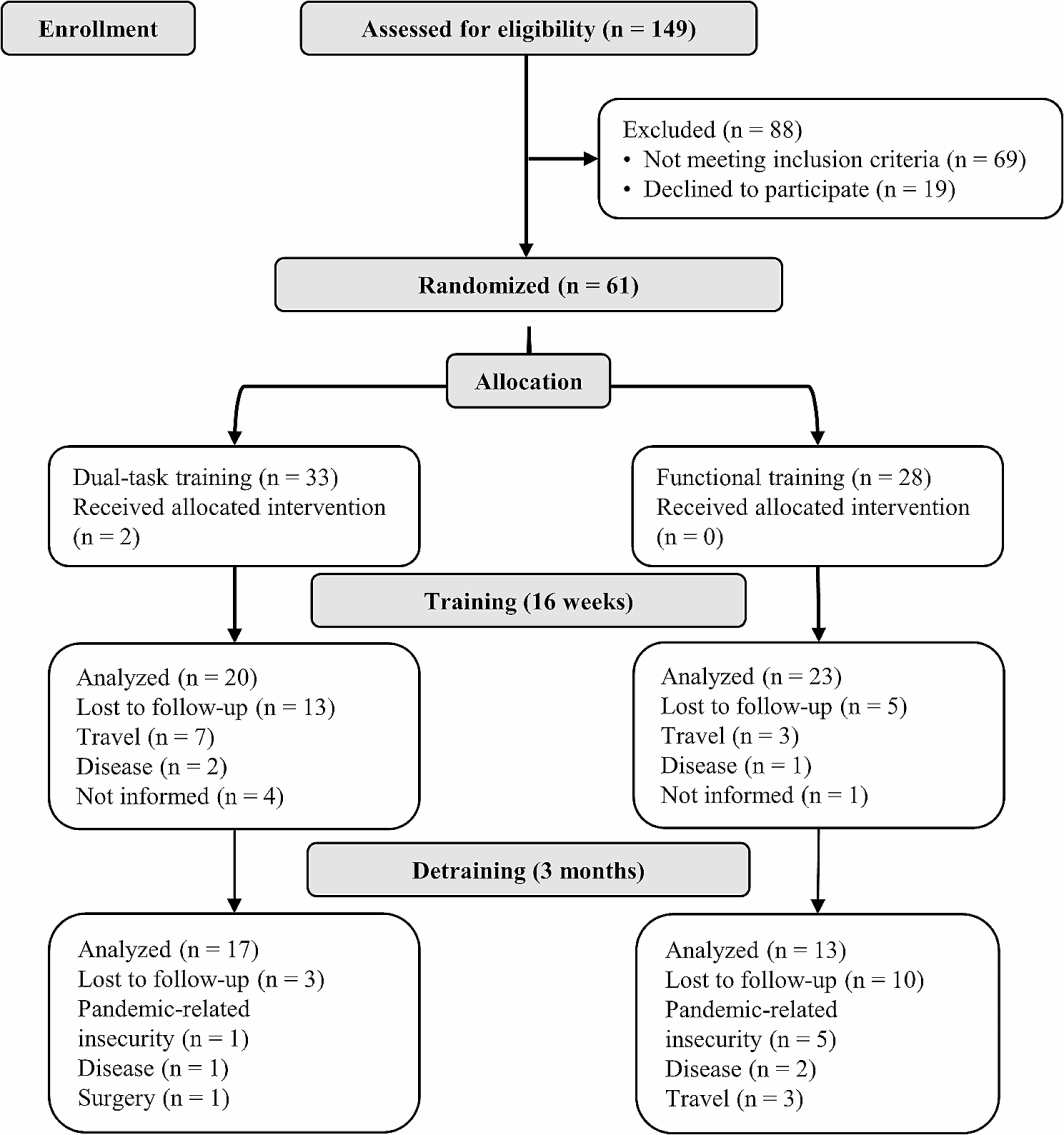
Flow diagram about the number of participants for each study phase

We initiated the study with 33 participants performing DT and 28 performing FT. After 16 weeks of intervention, 20 participants remained in the DT and 23 in the FT, and no adverse events occurred during the measures or training protocols. Additionally, during the training protocols, we identified an adherence of 72% for FT and 47% for DT (*p* = 0.023). However, the adherence did not significantly affect the dependent variables. Finally, following the three months of follow-up to evaluate the detraining effects, 17 and 13 participants remained in the DT and FT, respectively. Detailed information about the number of participants in each phase of the study and the reasons for dropouts is shown in Fig. 2.

Table 3 Shows the participants’ characteristics, demographic, and health information. No significant differences between groups were observed for any observed variables at the beginning of the study.

**Table 3:** Sociodemographic and medical characteristics of the participants in the study beginning

	Dual-task training (33)	Functional training (28)	Total (61)	
**Sociodemographic variables**	**Mean** **(95% CI lower - upper)**	**Mean** **(95% CI lower - upper)**	**Mean** **(95% CI lower - upper)**	***p***
**Age (years)**	68 (66–70)	67 (64–69)	67 (66–69)	0.396
**Body mass (kg)**	64.42 (59.72–69.13)	66.07 (62.14–70.01)	66.46 (64.03–68.89)	0.589
**Height (m)**	1.51 (1.49–1.53)	1.54 (1.52–1.57)	1.53 (1.52–1.55)	0.061
**BMI (kg/m** ^**2**^ **)**	28.02 (26.31–29.73)	27.66 (26.07–29.26)	28.27 (27.33–29.22)	0.757
**Medical history**	**Absolute frequency (%)**	**Absolute frequency (%)**	**Absolute frequency (%)**	**p**
**Hypertension**	20 (34.90)	13 (22.00)	33 (55.90)	0.085
**Dyslipidemia**	17 (28.80)	19 (32.20)	36 (61.00)	0.917
**Diabetes**	6 (10.20)	5 (8.50)	11 (18.60)	0.950
**Arthritis**	13 (22.00)	6 (10.20)	19 (32.20)	0.078
**Osteoporosis**	5 (8.50)	3 (5.10)	8 (13.60)	0.666

*Note* BMI = Body mass index; CI = Confidence interval; % = Relative frequency

Regarding the functional fitness results, we found a time effect for the PTS (χ^2^_(3)_ = 58.27; *p* < 0.001), SPP (χ^2^_(3)_ = 19.20; *p* < 0.001), GJST (χ^2^_(3)_ = 225.45; *p* < 0.001), and TUG (χ^2^_(3)_ = 51.10; *p* < 0.001). In addition, we detected a time x group interaction for the FSST (χ^2^_(3)_ = 8.16; *p* = 0.043) and W10m (χ^2^_(3)_ = 9.01; *p* = 0.029). Bellow, we detailed the founded effects for each variable separately.

**Fig. 3:**
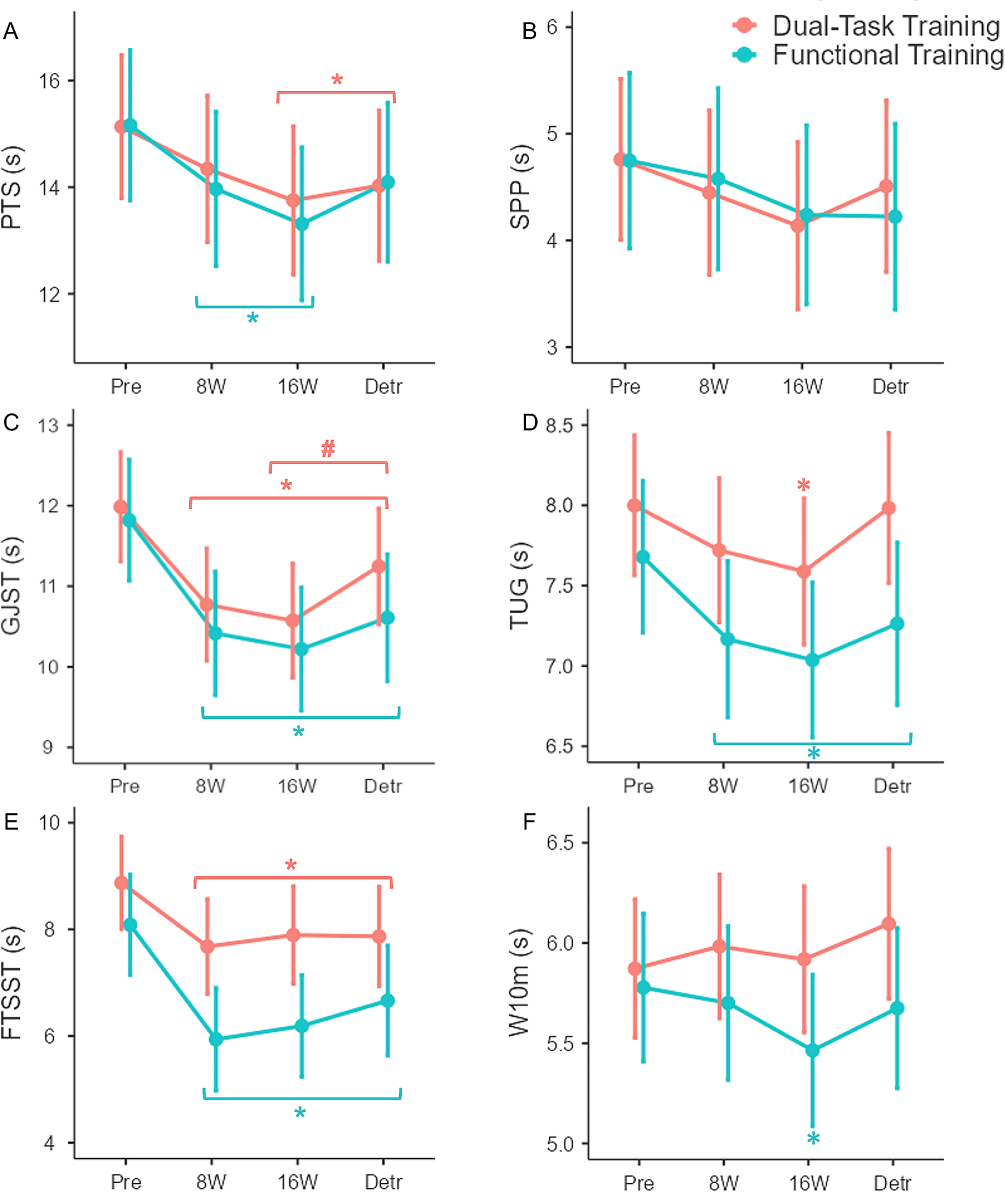
Functional fitness results after dual-task training and functional training in older women. *Note.* The beige line is related to Dual-task training, and the green line is to Functional training. Values showed as means ± 95% confidence intervals (CI). The effects are derived from generalized mixed models using Gamma distribution. Pre: pre-test, 8 W: 8 weeks, 16 W: 16 weeks, Detr: detraining * Indicates significant differences compared to the pre-test. Beige symbols are related to the Dual-task training and green symbols are related to the Functional training effects. PTS: put on and take off a t-shirt; SPP: standing-up from the prone position; TUG: timed up and go; FTSST: five times sit-to-stand test; GJST: gallon-jug shelf-transfer; W10m: 10 m walk test

In the PTS, the DT showed a small magnitude reduction after 16 weeks (d = 0.35; *p* < 0.001) and Detraining (d = 0.27; *p* = 0.022) compared to the pre-test. We also detected a small magnitude reduction in the time performance after 8 (d = 0.33; *p* = 0.005) and 16 weeks (d = 0.49; *p* < 0.001) in the FT compared to the pre-test values (Fig. 3A).

On the other hand, despite a time effect in the SPP between the 16 weeks (β = -0.565; *p* < 0.001) and detraining (β = -0.388; *p* = 0.011) compared to the pre-test, the post hoc comparisons did not indicate time differences between groups (Fig. 3B). In turn, the pairwise comparison for the GJST showed a reduced time to perform the test in both groups after 8 (DT: d = 0.64; *p* < 0.001; FT: d = 0.72; *p* < 0.001), 16 weeks (DT: d = 0.76; *p* < 0.001; FT: d = 0.82; *p* < 0.001), and Detraining (DT: d = 0.43; *p* < 0.001; FT: d = 0.64; *p* < 0.001) compared to the pre-test values (Fig. 3C). Additionally, the DT group showed a small magnitude time increase in the GJST performance after the detraining compared to the 16-week values (d = -0.40; *p* = 0.004).

Both groups reduced the time performance in the TUG. However, the DT demonstrated a small magnitude decrease only after 16 weeks (d = 0.34; *p* = 0.008) compared to the pre-test values. In turn, the FT induced a small to moderate magnitude reduction in the time performance after 8 (d = 0.42; *p* < 0.001), 16 weeks (d = 0.78; *p* < 0.001), and Detraining (d = 0.63; *p* = 0.040) compared to the pre-test (Fig. 3D).

For the FSST, both groups showed small to moderate magnitude reduction in the time performance after 8 (DT: d = 0.49; *p* < 0.001; FT: d = 0.89; *p* < 0.001), 16 weeks (DT: d = 0.41; *p* = 0.021; FT: d = 0.77; *p* < 0.001), and Detraining (DT: d = 0.42; *p* = 0.024; FT: d = 0.61; *p* < 0.001) (Fig. 3E). Furthermore, due to the interaction effect, we detected that the reduction produced by the FT was higher than the DT between the pre-test compared to 8 (β = 0.955; *p* = 0.012) and 16 weeks (β = 0.918; *p* = 0.020).

Finally, for the W10m, only the FT showed a small magnitude reduction in the time performance after 16 weeks (d = 0.32; *p* = 0.013) of intervention (Fig. 3F). Additionally, based on the interaction effect, we found that FT produce a larger time performance reduction than DT comparing the values after Detraining (β = 0.327; *p* = 0.031) and 16 weeks (β = 0.360; *p* = 0.006) with the pre-test values.

## Discussion

To the best of our knowledge, this is the first study to compare DT and FT on the functional fitness of older women. Also, this is a pioneer study investigating the detraining effects of DT on functional fitness. Our main finding was that FT and DT effectively improve the functional fitness of older women and maintain the benefits after three months of detraining. However, as we stated in our hypothesis, the FT showed earlier improvements than DT in the TUG test and was the only group to improve the W10m performance. Also, for all the measures, FT showed no difference between the performance after the post-test and detraining, keeping the differences between the pre-test and detraining. In contrast, DT performed worse in the GJST after the detraining than in the post-test. Therefore, FT and DT are similarly effective approaches in enhancing the functional fitness of older women, even though the FT seems more efficient in improving and maintaining older women’s functional fitness.

Regarding the upper limbs’ mobility based on the PTS, the DT promoted a small magnitude improvement in performance after 16 weeks and kept the adaptations after the detraining period compared to the pre-test. In turn, the FT induced a small improvement from eight weeks of intervention until 16 weeks, without differences after the detraining period compared to the pre-test. To the best of our knowledge, this is the first study to use the PTS test after a DT protocol, while a couple of studies show FT positive effects on this variable [29, 30]. Possibly, both training benefits occurred due to the emphasis on mobility in the initial part of the protocols based on the specificity principle [31]. Additionally, the faster FT adaptations could be related to the upper-limb power and strength exercises applied (e.g., medicine ball throws and battle rope), similar to other studies that used strength training [32, 33]. However, the small-magnitude improvement in both groups could be related to the absence of a more specific exercise similar to the tested action.

Both training protocols showed only a small magnitude, non-significant effect in the SPP. These results could be associated with the regular level of performance observed in both groups, according to Dantas et al. [34]. In this sense, Carrasco-Poyatos et al. [32] showed a large effect after an 18-week strength training protocol. However, the participants started the training protocol with low baseline performance, making greater training-related improvements possible. Another point was the absence of specific exercises exploring the ability to get up from the floor or for core muscles. De Matos et al. [30] showed positive effects using FT. However, their protocol dedicated a specific part to stimulating core muscles that play an important role in the SPP.

In the GJST, both training groups benefited from the eighth week of training until the detraining period. Our results corroborate previous studies showing the positive effects of FT [10, 29] and resistance training [35, 36] on this variable. These positive effects are likely related to the FT muscle power stimulus and the DT coordinative and reaction time activities emphasizing the upper limbs. However, interestingly, the DT showed a significant reduction after the detraining period compared to the values after 16 weeks of training, showing an adaptation loss after the training interruption. One possible reason for this finding is the quantity of stimulus for the upper limbs that was superior in the FT in the second and third parts of the training session. Anyway, despite the significant reduction observed after the interruption of the training protocol, the values remained better than the pre-test values.

The DT only promoted a small improvement in the TUG performance after 16 weeks of training, while the FT moderately improved the performance after eight weeks of training until the detraining period compared to the pre-test values. Our results for the DT training are similar to the literature showing positive effects of DT in the dynamic balance, as demonstrated by several studies applying different DT variations and durations [37, 38]. The competition theory of attention possibly explains that the DT-related benefits may be enhanced stimuli for the multi-task ability [12]. However, only the multi-task exercises without specific exercises for agility were not enough to produce benefits after eight weeks of intervention. On the other hand, the FT focused on the agility and power exercises produced TUG improvements earlier than DT, and this finding corroborates previous studies such as Aragão-Santos et al. [9] that found TUG improvements after four weeks of FT.

Both training groups improved the FTSST from the eighth week of training. However, the DT showed a small improvement in all measurements, while the FT promoted a large (8-weeks) and moderate magnitude improvement (16-weeks and detraining). These results corroborate with Sepúlveda-Loyola et al. [39], who showed a larger improvement in the FTSST using single-task (d = 1.0) than DT (d = 0.7) in older adults. The DT-related adaptations could be related to the inter- and intra-muscular coordination increase due to the balance activities. However, the absence of specific power and strength stimulus could have limited the magnitude of the adaptations. On the other hand, the FT applied a large component of power and strength focused on the lower limbs, which can explain the FT-related benefits consistent with previous studies [9, 10, 30].

Only the FT improved the performance in the walking ability measured using the W10m. Also, this result was only detected after 16 weeks of training. Similarly, De Matos et al. [30] only showed benefits in the W10m after 20 weeks of FT, and Ramos et al. [33] showed positive effects after 16 weeks of resistance or walking training. The strength and power stimulus applied in the FT could favor a better walking ability since muscle power and speed velocity are related [40]. However, the small magnitude improvement in the FT and the absence of effect in the DT group are possibly associated with the participants’ good initial performance level [30] and the absence of specific stimulus to lower limb power or walking speed in the DT group.

Despite our findings, this study had some limitations, such as the absence of a control group. However, understanding that regular physical exercise practice benefits older adults, it would be unethical to provide intervention for only one group. Besides, due to our previous findings on the FT benefits [9, 10], we chose to use the FT as the comparison group related to the DT. Another limitation was the absence of measures exploring dual-task situations to see if the DT could promote additional benefits compared to the FT due to the specificity principle. Nonetheless, our objective was to investigate if the DT could promote similar benefits to FT in older women’s functional fitness. Our sample presented some comorbidities, such as arthritis, that could influence the performance in the functional fitness assessments. Despite the comorbidities, the participants could perform the tests and follow the training protocols, showing feasibility. Also, the training stimuli can have indirectly improved the comorbidities, favoring the improvement in functional fitness. However, we recommend caution when interpreting our results, considering the sample characteristics to make inferences.

Another point was that the sample consisted only of older women, and the lower adherence showed in both groups, mainly in the DT. We chose older women due to the specificities of the aging process in women, such as menopause. It is worthwhile that adherence to the training protocols was a concerning point, considering the importance of regular practice in obtaining physical exercise benefits. Notwithstanding, in real-life scenarios, low adherence rates can be frequent due to physical limitations or illness. Thus, understanding the effects of our training protocols helps to evaluate if functional fitness has some benefits even in low adherence participants. In this sense, despite the lower adherence, we found positive effects for both FT and DT on functional fitness, and the adherence rate have not influenced the results when used as a covariate in the analysis.

This study has some strengths, such as its novelty, since, to the best of our knowledge, this is the first study to compare the DT and FT on the functional fitness of older women. Also, we provided valuable insights related to the benefits of DT on functional fitness as an alternative approach for people who are unable or do not want to perform FT. Both protocols promoted benefits in functional fitness despite the lower adherence, which is particularly relevant since the participants missed several sessions in real-life situations. Finally, even with the training interruption, both groups maintained the benefits they achieved in most of the variables compared to the pre-test. This shows the importance of a physical training period to maintain physical fitness when it is necessary to interrupt the practice, such as in the pandemic scenario [29].

## Conclusions

Sixteen weeks of DT and FT are similarly effective in improving the functional fitness of older women and maintaining their benefits even after three months of detraining. Therefore, both protocols can be used to improve the functional fitness of older women, but the FT provides faster adaptations than DT. Nonetheless, the FT is more effective than DT in improving upper limb mobility, lower limb muscle power, dynamic balance, and walking ability. Also, our DT training protocol can be an alternative for people who are unable or do not want to perform FT due to the external loads.

## Data Availability

The datasets used and analyzed during the current study are available from the corresponding author on reasonable request.

## Abbreviations

FT: Functional training
DT: Dual-task training
PTS: Put on and take off a t-shirt
SPP: Standing-up from the prone position
FTSST: Five times sit to stand test
TUG: Timed up and go
GJST: Gallon-jug shelf-transfer
W10m: 10 m walk test
BMI: Body mass index
CI: Confidence interval

## References

[1] Cunningham C, O’ Sullivan R, Caserotti P, Tully MA (2020). Consequences of physical inactivity in older adults: a systematic review of reviews and meta-analyses. Scand J Med Sci Sports.

[2] Li C, Ge S, Yin Y, Tian C, Mei Y, Han P (2023). Frailty is associated with worse cognitive functioning in older adults. Front Psychiatry.

[3] Monteleone P, Mascagni G, Giannini A, Genazzani AR, Simoncini T (2018). Symptoms of menopause - global prevalence, physiology and implications. Nat Rev Endocrinol.

[4] Chou M-Y, Nishita Y, Nakagawa T, Tange C, Tomida M, Shimokata H (2019). Role of gait speed and grip strength in predicting 10-year cognitive decline among community-dwelling older people. BMC Geriatr.

[5] Campitelli A, Paulson S, Vincenzo J, Glenn JM, Gills JL, Jones MD (2022). Sit-to-stand power across the lifespan: a cross-sectional analysis. J Aging Phys Act.

[6] Forman DE, Arena R, Boxer R, Dolansky MA, Eng JJ, Fleg JL (2017). Prioritizing functional capacity as a principal end point for therapies oriented to older adults with cardiovascular disease: a scientific statement for healthcare professionals from the American heart association. Circulation.

[7] Izquierdo M, Merchant RA, Morley JE, Anker SD, Aprahamian I, Arai H (2021). International exercise recommendations in older adults (ICSFR): expert consensus guidelines. J Nutr Health Aging.

[8] Mcleod JC, Stokes T, Phillips SM (2019). Resistance exercise training as a primary countermeasure to age-related chronic disease. Front Physiol.

[9] Aragão-Santos JC, Vasconcelos ABS, de Resende-Neto AG, Rodrigues LS, Silva N, de Silva L (2021). Functional and concurrent training do not impair immune function and improve functional fitness in postmenopausal women: a randomized controlled trial. Exp Gerontol.

[10] Rocha JN, de Vasconcelos S, Aragão-Santos ABS, de Resende-Neto JC, Monteiro AG, Nogueira MRP (2023). A single-set functional training program increases muscle power, improves functional fitness, and reduces pro-inflammatory cytokines in postmenopausal women: a randomized clinical trial. Front Physiol.

[11] La Scala Teixeira CV, Evangelista AL, Novaes JS, Da Silva Grigoletto ME, Behm DG (2017). You’re only as strong as your weakest link: a current opinion about the concepts and characteristics of functional training. Front Physiol.

[12] Khan MJ, Kannan P, Wong TW-L, Fong KNK, Winser SJ (2022). A systematic review exploring the theories underlying the improvement of balance and reduction in falls following dual-task training among older adults. Int J Environ Res Public Health.

[13] Herold F, Hamacher D, Schega L, Müller NG (2018). Thinking while moving or moving while thinking - concepts of motor-cognitive training for cognitive performance enhancement. Front Aging Neurosci.

[14] Tait JL, Duckham RL, Milte CM, Main LC, Daly RM (2017). Influence of sequential vs. simultaneous dual-task exercise training on cognitive function in older adults. Front Aging Neurosci.

[15] Gheysen F, Poppe L, DeSmet A, Swinnen S, Cardon G, De Bourdeaudhuij I (2018). Physical activity to improve cognition in older adults: can physical activity programs enriched with cognitive challenges enhance the effects? A systematic review and meta-analysis. Int J Behav Nutr Phys Act.

[16] Schulz KF, Altman DG, Moher D, Group CONSORT (2010). CONSORT 2010 Statement: updated guidelines for reporting parallel group randomized trials. BMC Med.

[17] Pinto TCC, Santos MSP, Machado L, Bulgacov TM, Rodrigues-Junior AL, Silva GA (2019). Optimal cutoff scores for dementia and mild cognitive impairment in the Brazilian version of the montreal cognitive assessment among the elderly. Dement Geriatr Cogn Dis Extra.

[18] Kreidler SM, Muller KE, Grunwald GK, Ringham BM, Coker-Dukowitz ZT, Sakhadeo UR (2013). GLIMMPSE: online power computation for linear models with and without a baseline covariate. J Stat Softw.

[19] Vale RGS, Pernambuco CS, Novaes JS, Dantas EHM (2006). Functional autonomy test: to dress and undress a sleeveless shirt (DUSS). Rev Bras Ci E Mov.

[20] Alexander NB, Ulbrich J, Raheja A, Channer D (1997). Rising from the floor in older adults. J Am Geriatr Soc.

[21] Goldberg A, Chavis M, Watkins J, Wilson T (2012). The five-times-sit-to-stand test: validity, reliability and detectable change in older females. Aging Clin Exp Res.

[22] Podsiadlo D, Richardson S (1991). The timed up & go: a test of basic functional mobility for frail elderly persons. J Am Geriatr Soc.

[23] Signorile JF, Sandler D, Ma F, Bamel S, Stanziano D, Smith W (2007). The gallon-jug shelf-transfer test: an instrument to evaluate deteriorating function in older adults. J Aging Phys Act.

[24] Peters DM, Fritz SL, Krotish DE (2013). Assessing the reliability and validity of a shorter walk test compared with the 10-meter walk test for measurements of gait speed in healthy, older adults. J Geriatr Phys Ther.

[25] La Scala Teixeira CV, Evangelista AL, Pereira PE, de Silva-Grigoletto A, Bocalini ME, Behm DS (2019). Complexity: a novel load progression strategy in strength training. Front Physiol.

[26] Behm DG, Sale DG (1993). Intended rather than actual movement velocity determines velocity-specific training response. J Appl Physiol.

[27] The jamovi project. (2023). jamovi (Version 2.3.28) [Computer Software]. Retrieved from https://www.jamovi.org.

[28] Cohen J (1988). Statistical power analysis for the behavioural sciences.

[29] Aragão-Santos JC, Pantoja-Cardoso A, Dos-Santos AC, Behm DG, de Moura TR, Da Silva-Grigoletto ME (2023). Effects of twenty-eight months of detraining imposed by the COVID-19 pandemic on the functional fitness of older women experienced in concurrent and functional training. Arch Gerontol Geriatr.

[30] De Matos DG, Mazini Filho ML, Moreira OC, DE Oliveira CE, Oliveira Venturini DE, Silva-Grigoletto GR et al. ME,. Effects of eight weeks of functional training in the functional autonomy of elderly women: a pilot study. J Sports Med Phys Fitness. 2017;57:272–7. 10.23736/S0022-4707.16.06514-2 27441915

[31] Reilly T, Morris T, Whyte G (2009). The specificity of training prescription and physiological assessment: a review. J Sports Sci.

[32] Carrasco-Poyatos M, Rubio-Arias JA, Ballesta-García I, Ramos-Campo DJ (2019). Pilates vs. muscular training in older women. Effects in functional factors and the cognitive interaction: a randomized controlled trial. Physiol Behav.

[33] Ramos AM, Marcos-Pardo PJ, Vale RG, de Vieira-Souza S, Camilo LM, de F B, Martin-Dantas EH (2022). Resistance circuit training or walking training: which program improves muscle strength and functional autonomy more in older women?. Int J Environ Res Public Health.

[34] Dantas EHM, Figueira HA, Emygdio RF, Vale RG (2014). Functional autonomy GDLAM protocol classification pattern in elderly women. Indian J Appl Res.

[35] Balachandran A, Martins MM, De Faveri FG, Alan O, Cetinkaya F, Signorile JF (2016). Functional strength training: seated machine vs standing cable training to improve physical function in elderly. Exp Gerontol.

[36] Buskard A, Zalma B, Cherup N, Armitage C, Dent C, Signorile JF (2018). Effects of linear periodization versus daily undulating periodization on neuromuscular performance and activities of daily living in an elderly population. Exp Gerontol.

[37] Brustio PR, Rabaglietti E, Formica S, Liubicich ME (2018). Dual-task training in older adults: the effect of additional motor tasks on mobility performance. Arch Gerontol Geriatr.

[38] Jardim NYV, Bento-Torres NVO, Costa VO, Carvalho JPR, Pontes HTS, Tomás AM (2021). Dual-task exercise to improve cognition and functional capacity of healthy older adults. Front Aging Neurosci.

[39] Sepúlveda-Loyola W, Maciel RPT, Teixeira DC, Araya-Quintanilla F, da Silva Junior RA, Santos SMS et al. Effect of dual-task training on clinical and biological factors related to Sarcopenia in older adults: a quasi-randomized controlled trial. 2022.

[40] Yee XS, Ng YS, Allen JC, Latib A, Tay EL, Abu Bakar HM (2021). Performance on sit-to-stand tests in relation to measures of functional fitness and sarcopenia diagnosis in community-dwelling older adults. Eur Rev Aging Phys Act.

